# 3D simulation of morphological effect on reflectance of Si_3_N_4 _sub-wavelength structures for silicon solar cells

**DOI:** 10.1186/1556-276X-7-196

**Published:** 2012-03-23

**Authors:** Yiming Li, Ming-Yi Lee, Hui-Wen Cheng, Zheng-Liang Lu

**Affiliations:** 1 Parallel and Scientific Computing Laboratory, Department of Electrical Engineering, National Chiao Tung University, 1001 Ta-Hsueh Road, Hsinchu 300, Taiwan

## Abstract

In this study, we investigate the reflectance property of the cylinder, right circular cone, and square pyramid shapes of silicon nitride (Si_3_N_4_) subwavelength structure (SWS) with respect to different designing parameters. In terms of three critical factors, the reflectance for physical characteristics of wavelength dependence, the reflected power density for real power reflection applied on solar cell, and the normalized reflectance (reflected power density/incident power density) for real reflectance applied on solar cell, a full three-dimensional finite element simulation is performed and discussed for the aforementioned three morphologies. The result of this study shows that the pyramid shape of SWS possesses the best reflectance property in the optical region from 400 to 1000 nm which is useful for silicon solar cell applications.

## 1. Introduction

Silicon solar cell is one of the promising renewable energy technologies in order to relieve the impact of the climate change. In semiconductor-based solar cells, electron-hole pairs are generated through absorption of impinging photons. Due to high refraction index of semiconductor materials, especially silicon, the incident sunlight power is largely reflected back, resulting in the reduction of light absorption and poor energy conversion efficiency. Antireflection coating (ARC) is mounted over absorption layers, resulting in three effects: (a) reduction in surface reflection, (b) increase in light absorption due to an increase in optical path length by diffraction, and (c) enhancement of internal reflection that reduces the amount of escaping light. Based on the theory of impedance matching, single layer (SLR) and multilayer of ARC are proposed for reduced reflectance property; however, the resulting reflectance spectra meet the demand only within a narrow spectral domain. Subwavelength structure's (SWS) dimensions are much smaller than the wavelengths of light; therefore, using ARC on the surface of silicon solar cells can substantially reduce the reflectivity and improve the capability of light trapping. It thus will achieve the enhanced efficiency according to our recent both numerical and experimental studies [1-3]. Compared with silicon solar cell with a SLAR, the efficiency of silicon solar cell with Si_3_N_4 _SWS is promising among various ARC layers in our recent work [4]. A rigorous coupled-wave analysis (RCWA) [1,5-7] has been reported to estimate the reflectance of Si_3_N_4 _SWS by approximating structural shapes with partitioned uniform homogeneous layers. RCWA is an exact solution of Maxwell's equations for the electromagnetic diffraction by grating structures which is generally applicable for 2D plane with 1D periodicity; however, RCWA may suffer numerical difficulties in presence of evanescent orders and it requires a large amount of calculation for retaining several diffraction orders. These factors limit flexible application of RCWA; in particular, for 3D problems with non-azimuthally symmetric structural shapes. Numerical simulation of 3D morphological effect on reflectance property has not been studied yet. Therefore, a full 3D finite-element (FE) analysis of Si_3_N_4 _SWS will be an interesting examination for quantitative understanding of the reflectance property.

In this study, 3D FE simulation for the reflectance of Si_3_N_4 _SWS with three types of structural shapes, the cylinder, the right circular cone, and the square pyramid shapes, is conducted with respect to different geometry parameters and lighting angles for quantitative understanding of reflectance property. First, proper selection on the boundary conditions can alleviate the computational load from simulating a holistic ARC. The reflectance of Si_3_N_4 _SWS on the silicon substrate is thus simulated using the 3D finite element method (FEM); consequently, in terms of three critical factors, the reflectance for physical characteristics of wavelength dependence, the reflected power density for real power reflection applied on solar cell, and the normalized reflectance (reflected power density/incident power density) for real reflectance applied on solar cell are calculated and discussed for the aforementioned three morphologies. The analysis of reflectance spectrum with wide-angle incidences of electromagnetic wave and the average reflectance with various heights are presented. Besides, according to our recent study, which presented the optimal design parameters of Si_3_N_4 _SWS based on RCWA [4], numerical verification and comparison is accomplished following the discussion. The engineering findings of this study show that the pyramid shape of SWS possesses the best reflectance property in the optical region from 400 to 1000 nm which is useful for silicon solar cell applications.

This rest of the article is organized as follows. In Section 2, we show the computational structure and model. In Section 3, we report the results and discussion. Finally, we draw conclusions and suggest future work.

## 2. The SWS and optical model

**Figure 1:**
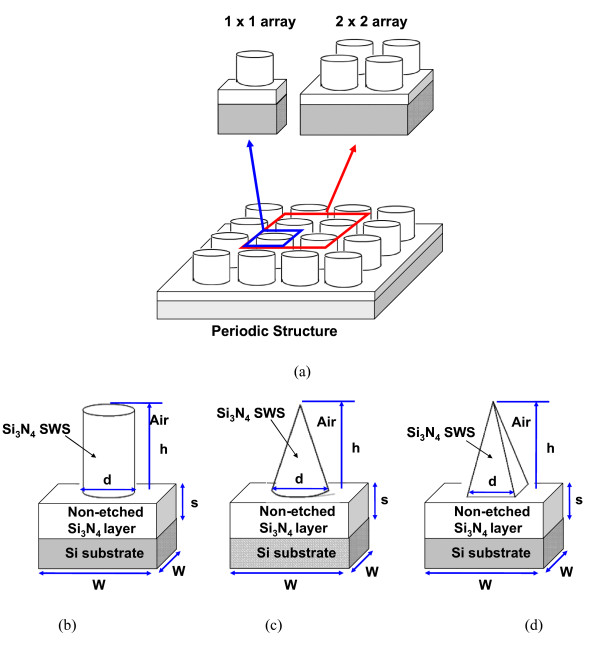
**(a) Plot of the periodic structure of Si_3_N_4 _SWS with 1 × 1 and 2 × 2 arrays as unit cell**. 3D schematic plots of the examined **(b) **cylinder-, **(c) **right circular cone-, and **(d) **square pyramid-shaped structure, respectively.

Based upon our experimental characterization, Figure 1a illustrates a periodical structure of Si_3_N_4 _SWS which is used in our 3D FE simulation without loss of generality. We study Si_3_N_4 _SWS with the cylinder, the right circular cone, and the square pyramid shapes, as shown in Figure 1b-d, respectively. With a constant volume, the diameter of cylinder- and right circular cone-shaped Si_3_N_4 _SWS and the edge length of square pyramid are 130 nm, the heights (h) of the etched part of Si_3_N_4 _SWS are 200, 600, and 471.3 nm, the height (s) of the non-etched part is 70 nm, and the base (W) of a unit cell is 200 nm [4]. Note that the thickness of Si substrate is given 600 nm. Note that all structural parameters are adopted from our experimental studies [2-4,8]. Throughout the article, we consider time-harmonic fields assuming a time-dependence in *e^-jωt^*. The diffraction problem is governed by the well-known Maxwell equations

(1) $$\text{∇}\times \bar{E}=-j\omega \mu \bar{H},$$

(2) $$\text{∇}\times \bar{H}=\bar{J}+j\omega \varepsilon \bar{E},$$

(3) $$\text{∇}\cdot \bar{E}=\rho /\varepsilon ,$$

and

(4) $$\text{∇}\cdot \bar{B}=0,$$

where *E *and *D *are electric field intensity and flux density, *H *and *B *are magnetic field intensity and flux density, *λ *is the corresponding frequency to the wavelength *λ, J*, and ρ are current density and charge density, *ε *is electric permittivity, *μ *is magnetic permeability. A repeated pattern is applicable to use periodic boundary conditions, thus the Floquet theorem is adopted to simulate the boundary condition of periodic structure. Floquet theorem asserts that the analysis region can be reduced significantly in one periodicity cell to characterize the propagation property. The electric fields in periodic structure are related as follows:

(5) $${Ē}_{2}\left(\bar{r}\right)={Ē}_{1}\left(\bar{r}+\bar{L}\right)={Ē}_{1}\left(\bar{r}\right)e^{-j\theta },$$

where *r *is position vector, *L *is the distance between the periodic boundaries, and *θ *is a phase factor determined by wave vector *k *and *L*:

(6) $$\theta =\bar{k}\cdot \bar{L,}$$

The polarization of transverse electric (TE) mode, in which the electric field is normal to the direction of wave propagation, is excited as the normal incident light source with wavelengths sweeping from 400 to 1000 nm. The bottom region of Si substrate is assigned as perfect matched layer in avoidance of reflected wave. The refraction index of Si_3_N_4 _is 2.05, and the refraction index of Si is frequency dependent with the relation [1]:

(7) $$n_{Si}=\sqrt{\varepsilon +\frac{A}{\lambda^{2}}+\frac{B\lambda_{1}^{2}}{\left(\lambda^{2}-\lambda_{1}^{2}\right)}},$$

where *λ *is the incident wavelength, *A *= 0.939816, *B *= 8.10461 × 10^-3^, λ_1 _= 1.1071 μm, and ε = 11.6858. The calculation settings of reflectance were reported and can be found in our recent studies [1,4].

## 3. Results and discussion

**Figure 2:**
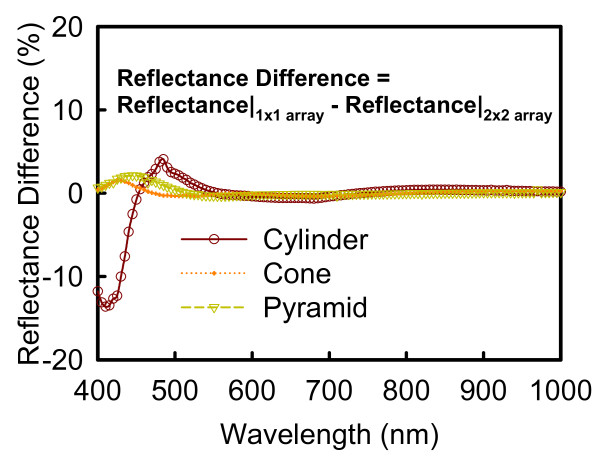
**Plot of the difference of reflectance spectrum of Si_3_N_4 _SWS with the cylinder-, the right circular cone-, and the square pyramid-shaped structures as well as two different periodical configurations: 1 × 1 (solid line) and 2 × 2 arrays (dashed line) in the 3D FEM simulation**.

**Figure 3:**
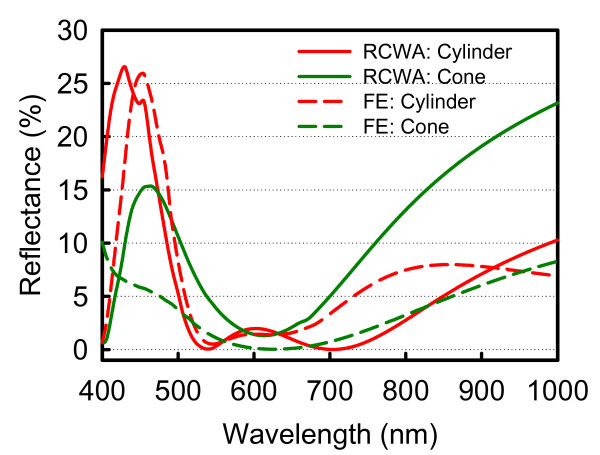
**Comparison of the reflectance spectra for the cone- and cylinder-shaped Si_3_N_4 _SWS calculated by RCWA and 3D FE analysis with the same design parameters**.

In order to examine the effect of Floquet boundary condition in 3D FE analysis, as shown in Figure 2, we compare the difference between the simulated unit cells of 1 × 1 and 2 × 2 array of Si_3_N_4 _SWS. We find at the wavelengths above 600 nm, the reflectance of 1 × 1 array of Si_3_N_4 _SWS as unit cell is almost consistent with unit cell of 2 × 2 array, meanwhile insignificant discrepancy occurs at wavelengths shorter than 600 nm. Based on this consequence, it is enable us to do simulation with more computational efficient using 1 × 1 array of Si_3_N_4 _SWS as a simulated unit cell with engineering acceptable accuracy. According to our recent RCWA work [4], the reflectance spectra are first plotted in Figure 3 using the optimal design parameters [1,4]. Also, the spectra calculated by a full 3D FE analysis with the same design parameters are indicated by dashed lines. For the cylinder-shaped Si_3_N_4 _SWS, the reflectance spectra for RCWA and FE analysis are similar, but not agreed for the cone-shaped Si_3_N_4 _SWS due to existing evanescent orders along the top of structures. This comparison confirms the importance of 3D FEM simulation which is beyond the RCWA approach [4].

**Figure 4:**
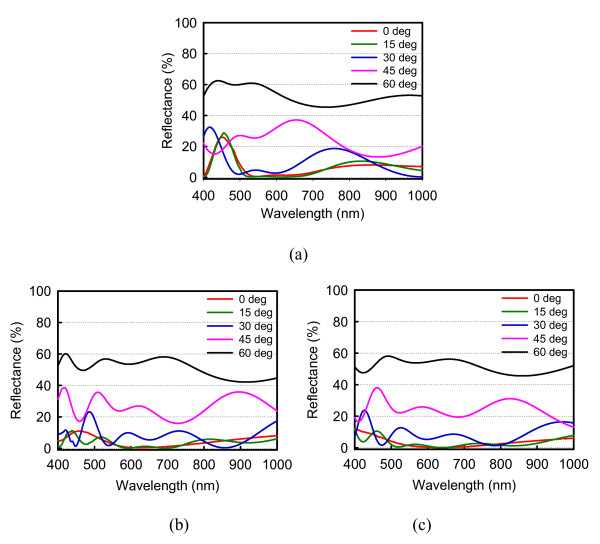
**Plots of the reflectance spectrum for the **(a) **cylinder- **(b) **circular-cone-, **(c) **and square-pyramid-shaped Si_3_N_4 _SWS with incident angles of 0°, 15°, 30°, 45°, and 60°**.

**Figure 5:**
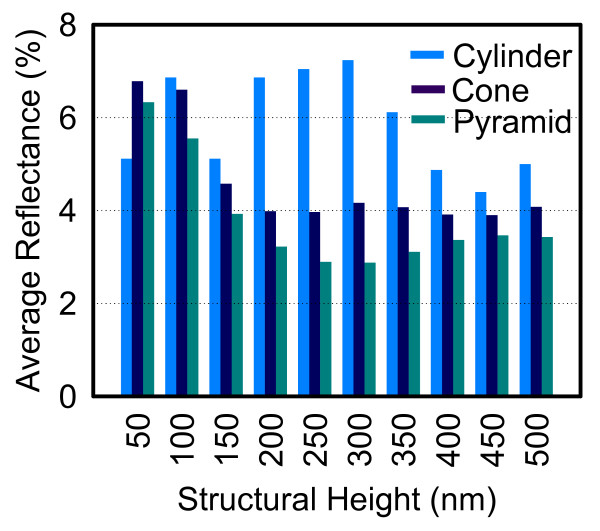
**Plot of the average reflectance among the studied three shapes of Si_3_N_4 _SWS with heights varying from 50 to 500 nm**.

**Figure 6:**
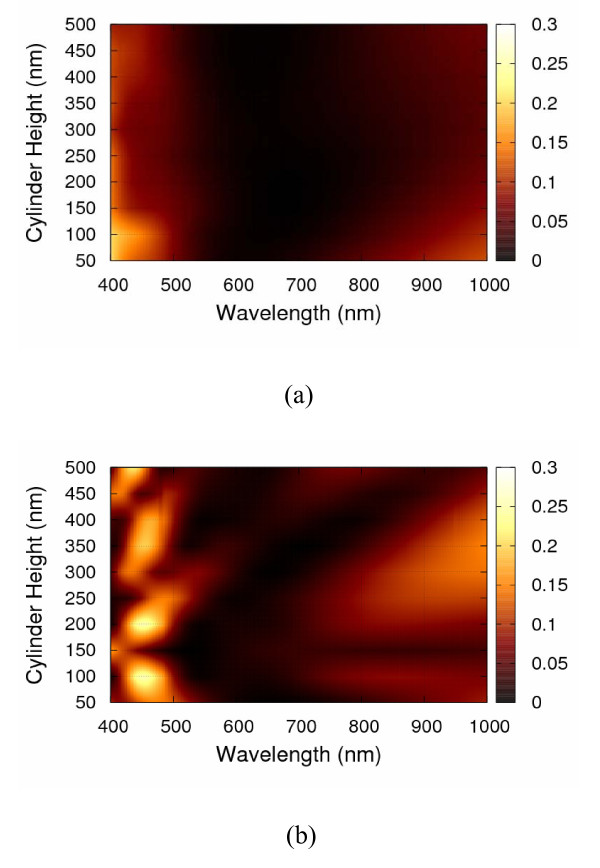
**3D view for the height effect on the reflectance with respect to different wavelength**. **(a) **The pyramid-shaped Si_3_N_4 _SWS has lower reflectance and less sensitivity on structure height in comparison with **(b) **the cylinder-shaped Si_3_N_4 _SWS.

**Figure 7:**
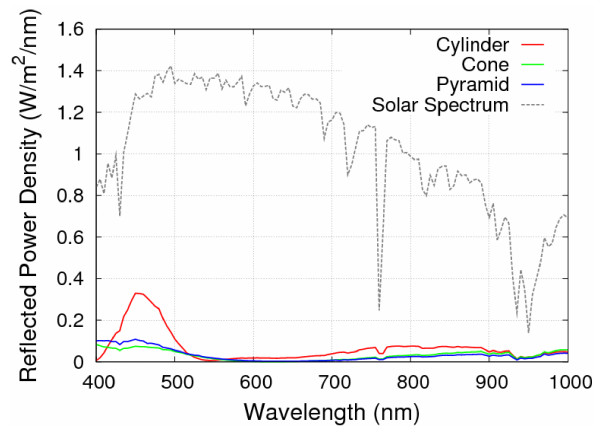
**Plot of the reflected power density among three different shapes**.

Figure 4a-c shows the reflectance spectra with incident angles of 0°, 15°, 30°, 45°, and 60° for the cylinder-, right circular cone-, and square pyramid-shaped Si_3_N_4 _SWS, respectively. For the normal incidence case, the lowest average reflectance among three structural shapes is 3.47% of square pyramid-shaped structure. The others are 6.86 and 4.42% for the cylinder- and the right circular cone-shaped Si_3_N_4 _SWS, respectively. Meanwhile, as shown in Figure 4a-c, one can observe that the reflectance increases significantly with larger incident angles, resulting in average reflectance beyond 50%. Table 1 summarizes the average reflectance for various incident angles. Height effect on average reflectance of Si_3_N_4 _SWS at normal incident angle with *d *= 130 nm and *s *= 70 nm is also calculated, as shown in Figure 5. The resulting average reflectance of pyramid-shaped Si_3_N_4 _SWS nearly keeps lowest in comparison with the cylinder- and the right circular cone-shaped Si_3_N_4 _SWS as the structural height is ranging from 50 to 500 nm. Figure 6 shows the reflectance dependence on the structural height and wavelength. The pyramid-shaped Si_3_N_4 _SWS has lower reflectance and less sensitivity on structure height in comparison with the cylinder-shaped Si_3_N_4 _SWS. Hence, the impact of process variation of structure height on solar cell performance is smaller for pyramid-shaped Si_3_N_4 _SWS. Based on solar spectrum at the sea level revealed in American Society for Testing and Materials (ASTM) Standard Tables for Reference Solar Spectral Irradiances: Direct Normal and Hemispherical 37 Tilted Surface [9], we further estimate the reflected power density (W/m^2^/nm) defined by reflectance times incident power density, as shown in Figure 7. The higher reflected power density of cylinder-shaped Si_3_N_4 _SWS (red line) indicates the less efficiency in the solar cell application. Therefore, normalized reflectance defined as

(8) $$R_{\text{norm}}\equiv \frac{\text{Reflected}\;\text{power}\;\text{density}}{\text{Incident}\;\text{power}\;\text{density}}$$

**Table 1:** Summary of the average reflectance of Si_3_N_4 _SWS with various incident angles

Average reflectance (%)	0°	15°	30°	45°	60°
Cylinder shape	6.86	6.70	9.95	23.13	52.78
Circular cone shape	4.42	3.64	7.79	26.68	50.98
Square pyramid shape	3.47	3.15	8.88	24.40	51.44

reveals the real power efficiency applied in the solar cell application. Figure 8 shows the normalized reflectance for the cylinder-, right circular cone-, and square pyramid-shaped Si_3_N_4 _SWS, respectively. The square pyramid-shaped Si_3_N_4 _SWS again shows the lowest normalized reflectance 3.13% while the cylinder- and the right circular cone-shaped Si_3_N_4 _SWSs have 6.66 and 4.12%, respectively.

**Figure 8:**
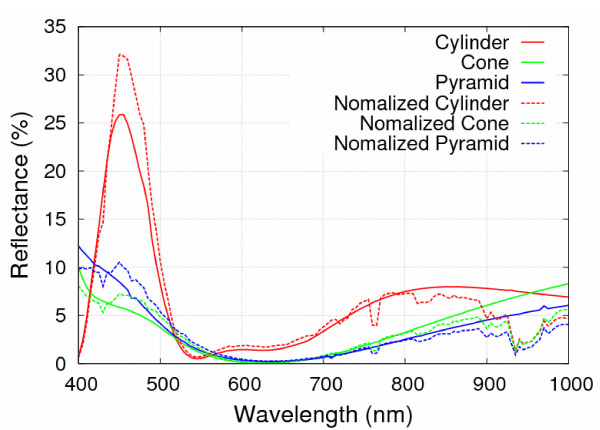
**Plot of reflectance with and without considering incident solar spectrum at sea level**.

## 4. Conclusions

In this study, the reflective property of unit cell with a validated Floquet boundary condition has been calculated using a full 3D FE simulation. Considering various incidence angles and height effect on three experimentally observed structural shapes of Si_3_N_4 _SWS, we have concluded that the pyramid-shaped Si_3_N_4 _SWS has best reflective property in the analysis of morphological effect. Compared with the results of RCWA, the reflective property calculated by the full 3D FEM is significantly deviated from the results from RCWA, giving the hint that a detailed and comprehensive methodology is dispensable for the design of Si_3_N_4 _SWS. The results of computed reflectance, reflected power density, and normalized reflectance have shown that the pyramid shape of SWS may have the best reflectance property in the optical region from 400 to 1000 nm which is useful for silicon solar cell applications. The optimized pyramid-shaped Si_3_N_4 _SWS is currently under plan for implementation with silicon solar cells.

## Abbreviations

3D: three-dimensional; ARC: antireflection coating; FEM: finite element method; RCWA: rigorous coupled-wave analysis; Si_3_N_4_: silicon nitride; SLAR: single layer; SWS: subwavelength structure.

## Competing interests

The authors declare that they have no competing interests.

## Authors' contributions

M-YL, H-WC, and Z-LL performed the numerical simulation and data analysis, YL conducted whole study including manuscript preparation. All the authors read and approved the final manuscript.
